# Overview of Rapid Detection Methods for *Salmonella* in Foods: Progress and Challenges

**DOI:** 10.3390/foods10102402

**Published:** 2021-10-11

**Authors:** Minglu Wang, Yilun Zhang, Fangyuan Tian, Xiaoyu Liu, Shuyuan Du, Guocheng Ren

**Affiliations:** College of Life Science, Shandong Normal University, Jinan 250014, China; w_ml2020@163.com (M.W.); zhangyilun0@163.com (Y.Z.); fanguyuan@163.com (F.T.); liuxiaoyu20200145@163.com (X.L.); dusy@sdnu.edu.cn (S.D.)

**Keywords:** *Salmonella*, food contaminant, recognition element, bioreceptor, rapid detection method

## Abstract

*Salmonella* contamination in food production and processing is a serious threat to consumer health. More and more rapid detection methods have been proposed to compensate for the inefficiency of traditional bacterial cultures to suppress the high prevalence of *Salmonella* more efficiently. The contamination of *Salmonella* in foods can be identified by recognition elements and screened using rapid detection methods with different measurable signals (optical, electrical, etc.). Therefore, the different signal transduction mechanisms and *Salmonella* recognition elements are the key of the sensitivity, accuracy and specificity for the rapid detection methods. In this review, the bioreceptors for *Salmonella* were firstly summarized and described, then the current promising *Salmonella* rapid detection methods in foodstuffs with different signal transduction were objectively summarized and evaluated. Moreover, the challenges faced by these methods in practical monitoring and the development prospect were also emphasized to shed light on a new perspective for the *Salmonella* rapid detection methods applications.

## 1. Introduction

The contamination of bacteria is a problem that cannot be ignored in food production and processing, and which may cause inestimable health damages to consumers [1,2,3,4]. Every year, more than half of the food-borne diseases in the world are caused by food-borne pathogens. As one of the major food-borne pathogens, *Salmonella* caused hundreds of thousands of deaths every year [5,6,7]. In the United States, the annual economic losses caused by *Salmonella* exceed $3 billion, which does not include the cases that were not reported [8,9,10].

*Salmonella*, a species of Gram-negative bacteria with more than 2500 serotypes, is responsible for a food-borne illness in humans and animals. Meanwhile, it can easily contaminate meat, eggs, milk and other foods, which leads to its strong transmission and difficulties of prevention and control [11,12]. Moreover, only one colony-forming unit (CFU) of *Salmonella*, the extremely low pathogenicity limit, can cause human infection, which put forward higher requirements for the prevention and control of food *Salmonella* contaminants [13]. Considering the serious threat of *Salmonella* to human health, the effective identification and rapid detection of *Salmonella* is of importance to prevent outbreaks of food-borne disease [14,15,16]. Nowadays, the conventional culture method is known as the “gold standard method” for *Salmonella* detection in food enterprises and testing companies. However, the long waiting time of the standard method is an enormous drawback for the immediate detection of *Salmonella*, which would greatly affect the transportation of foods to the markets [17]. With the development of molecular biology, a variety of specific *Salmonella* bioreceptors, such as antibody and aptamer, have been obtained [18,19,20], and more and more rapid detection methods of *Salmonella* based on specific bioreceptors have also been presented recently, which is of innovative significance for the real-time *Salmonella* detection in foods [21,22,23,24,25].

**Figure 1:**
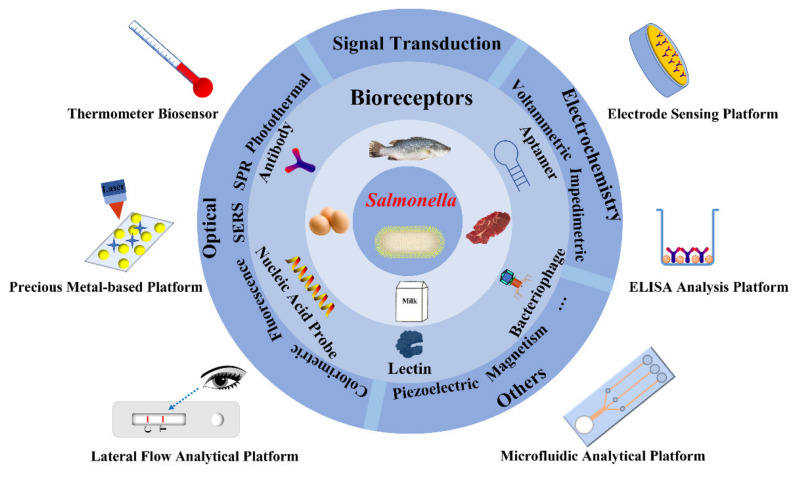
Overview of the bioreceptors and rapid detection methods for *Salmonella*.

In this review, the bioreceptor of *Salmonella* which is the key factor affecting the sensitivity and specificity of rapid detection was described in detail as shown in Figure 1. Then, some popular *Salmonella* rapid detection methods applied in foods were emphatically descripted. Besides, the current trends of *Salmonella*’s rapid detection methods, the existing challenges and the application perspectives were also reviewed and discussed.

## 2. Gold Standard Method

Most countries and regions have set strict standards to ensure the absence of *Salmonella* in foodstuffs (as in China and the European Union), which shows the necessity of a sensitive detection of *Salmonella* [26,27]. Nowadays, microbial culture is still the gold standard strategy for the identification of *Salmonella* in many countries [28]. This method usually employs selective and differential media containing different nutrients and specific pH conditions to isolate *Salmonella* [13,29]. To achieve an accurate *Salmonella* identification in samples with a small amount and low residual, it is usually necessary to enrich the bacteria in the enrichment medium, which prolongs the detection time. Including subsequent bacterial counting and biochemical experiments, the whole detection process may take at least 5 days. Moreover, the type of testing applied is dictated by the existing legal requirements that food business operators should conform to, but the long waiting time is painful for products like fresh foods that need to be rapidly served to the market [30]. Therefore, these practical requirements also emphasize the need for the development of ideal rapid *Salmonella* detection methods in foods.

## 3. Bioreceptors for *Salmonella*

The rapid detection of *Salmonella* could not be separated from its effective identification. Recognizing the *Salmonella* molecule is one of the most important conditions for its rapid detection, which affects the efficiency of the separation and the direct signal output. Bioreceptor is a general term for the biological recognition molecule that can recognize the target [31,32,33]. After sorting out the published articles on *Salmonella* detection in recent years, the commonly used bioreceptors were summarized, including antibody, aptamer, nucleic acid probe, bacteriophage and lectin. The comprehensive comparison of the five bioreceptors is presented in Table 1.

### 3.1. Antibody

Antibody is a kind of immunoglobulin (Ig) generally produced by the mammalian immune system. IgG, one kind of Ig, has a strong affinity for its target, which is commonly used as a bioreceptor to identify the target in food contaminants detection [34,35]. The typical IgG molecule possesses a basic “Y” structure and consists of two heavy chains and two light chains, including the fragment crystallizable (FC) region for immune response activation and the fragment antigen-binding (Fab) region for antigen recognition.

The carboxyl group (-COOH) on the lower side of the Fab site is conducive to the fixation of the antibody on the surface of the sensor to realize the detection of the recognition signals. Moreover, the antibody exhibits a high selectivity to antigens and a strong anti-interference ability due to the unique epitope. Therefore, the antibody has been recognized as the standard recognition factor of commercial rapid detection products used in food safety detection, especially in immunochromatographic lateral flow strips and the enzyme-linked immunosorbent assay (ELISA) [36,37,38]. However, antibodies need to be produced by mammals or animal cells, which greatly slows down their production and leads to their high price. Furthermore, the activity of antibodies is vulnerable to organism and external environments, which weakens their stability [39,40]. Therefore, there are also some limitations for the powerful antibody.

Following the good performance of the antibody, a new type of antibody, called nanobody, is being developed to reduce the limitations of the traditional antibody. The nanobody, with an affinity to the antigen similar to that of the antibody, can be produced abundantly and has an extremely small size (12–15 kDa), so that it can bind to epitopes of antigens more conveniently than traditional antibodies (150–160 kDa) [41]. Although the nanobody has many advantages, the preparation of immune libraries is still difficult, and the development for the detection of food contaminants is still immature. However, nanobodies have been developed by pioneers to detect *Salmonella*. He et al. prepared the *Salmonella* nanobody library and verified the feasibility of its application in *Salmonella* detection by a mature ELISA, which also laid the foundation for the further development of the nanobody [42].

### 3.2. Aptamer

Broadly speaking, aptamers are single-stranded nucleotides or short-peptide molecules. Only the nucleic acid aptamer is introduced here. Aptamer, a single-stranded DNA or RNA selected by Systematic Evolution of Ligands by Exponential Enrichment (SELEX, a selection technology of nucleic acid aptamer) in vitro, is considered as a good substitute for the antibody and has been used as bioreceptor in various biosensors for the detection of food contaminants [19,43]. The short aptamer can form a stable and complex three-dimensional structure to recognize antigens of *Salmonella* specifically. Meanwhile, because of the aptamer’s miniaturization, it can also recognize special sites which are difficult to recognize by traditional antibodies [44].

Aptamer, a non-immunogenic recognition molecule with thermal stability and chemical modifiability, is unchallenged by antibodies and can be synthesized in large quantities in vitro at a low cost [45]. Aptamers are employed for the detection of inorganic molecules, proteins, cells and other molecules by transmitting the signal changes of conformational aptamers which combined with the target. One of the powerful obstacles in the aptamer sensing strategy is the immobilization of the aptamer onto the surface of the sensor while ensuring the proper functioning of the aptamer analysis. In general, the direct absorption aptamer by biotin/streptavidin binding is a suitable choice for the immobilization of aptamers on the carrier surface, which has the least impact on the specificity and affinity of the aptamer. Based on this, the special aptamer-based biosensors (aptasensors), including those used for *Salmonella* detection, have become an independent detection platform. In Bayraç’s study, for example, the aptamer of *Salmonella Enteritidis* was selected by Cell-SELEX, and the colorimetric detection method was established by a capillary tube, which proved the feasibility of the aptamer for the naked-eye detection of *Salmonella* for the first time, paving the way for the development of the subsequent method [18]. The astonishing performance of the aptamer in the detection field has really won the favor of many people.

### 3.3. Nucleic Acid Probe

Macromolecular nucleic acid sequences are composed of two kinds of nucleotides usually present in cells: deoxyribonucleic acid (DNA), which stores genetic material, and ribonucleic acid (RNA), which produces proteins. The genetic sequences of different strains or individual microorganisms are specific. Thus, the nucleic acid sequence can provide biological information on food-borne pathogens, and then the determination of its sequence is critical in the detection of food-borne pathogens. The nucleic acid probe is the complementary sequence of the special commentary strand of *Salmonella*, which can be cheaply synthesized and is relatively stable, like an aptamer. Through its high affinity and the specificity of base pairing, the nucleic acid probe can identify the presence of the bacteria and realize the transmission and measurement of special recognition signals [46,47]. Generally, nucleic acid probes rely on the signals generated by special groups that were modified with the signal molecule (such as fluorophore groups), and beyond that, through a modification on the surface of the sensor by the nucleic acid probe, which outputs the detection signals. However, the nucleic acid probe is always limited by the recognition of the target nucleic acid sequence, and the signal sensing of the methods generally depends on gene amplification and laborious gene extraction.

### 3.4. Bacteriophage

Bacteriophage-based biosensors are also the research focus of *Salmonella* detection in recent years [48]. A bacteriophage, a kind of virus, is a powerful bioreceptor for bacterial recognition, which can specifically infect living bacteria with high host selectivity. A bacteriophage can only replicate in viable hosts, which makes it a potential tool for distinguishing living and dead bacteria. The identification of living *Salmonella* is interesting and was demonstrated by Fernandes et al. They successfully distinguished viable, dormant and dead *Salmonella Enteritidis* by using a bacteriophage as a bioreceptor [49]. It is of great significance for *Salmonella* control in foods, by identifying the contaminations of viable and viable but non-culturable bacteria. Various biosensors have been developed based on a variety of reported *Salmonella* bacteriophages (including M13, PRD1, P22), which benefit from the strong resistance of bacteriophages to harsh conditions. The bacteriophage-based *Salmonella* detection platforms have been successfully commercialized because of their good detection results [50,51,52]. However, along with the good prospect of phages, the lysis of the *Salmonella* extracted from phages will lead to the decrease of the capture efficiency and the reduction or loss of recognition signals, which is also a key challenge faced by researchers in this field. Nevertheless, the bacteriophage-based detection method is still the breakthrough for the rapid and accurate identification of living bacteria, and the more innovative bacteriophage biosensing strategy would be an expected demonstration in the future.

### 3.5. Lectin

The O-antigen, constituting the lipopolysaccharide (LPS) on the cell wall of Gram-negative bacteria, can be used for strain distinction. The LPS O-antigens can exhibit a high specific lectin binding affinity that indicates the recognition ability of lectin to *Salmonella*. As a non-enzyme and non-antibody protein, lectin has a higher stability, but a lower price compared with antibodies. At present, biosensors using lectins as recognition molecules have emerged one after another, and the more commonly used lectins are concanavalin A and wheat germ agglutinin [53]. Dao et al. enriched *Salmonella* by using concanavalin A as the recognition molecule, combined with microfluidic technology and isothermal amplification for target DNA detection, with the limit of detection (LOD) as low as 5 CFU/mL [54]. Then, much work has been devoted to optical and electrochemical bacterial biosensors utilizing lectin as a recognition element. With the continuous development and application of nanomaterials, the detection methods combined with various materials are also booming. It is believed that lectin will have a promising role in the field of *Salmonella* detection.

## 4. Rapid Detection Methods of *Salmonella* in Foods

With the manufacture of advanced instruments and the continuous development of nanomaterials, various rapid detection methods for *Salmonella* are constantly being developed, based on the above bioreceptors. However, a different signal transduction also determines the complexity and sensitivity of the rapid detection method. Here, rapid detection methods of *Salmonella* based mainly on optical sensing and electrochemical identification methods were introduced.

### 4.1. Optical Sensing

Optical sensing, one of the fastest developing methods of *Salmonella* detection, converts the biological and chemical reactions that occur between bioreceptors and targets into optical signals through transducers or detection instruments [55]. Optical sensing mainly includes colorimetry, fluorescence analysis, surface-enhanced Raman spectral (SERS) detection, surface plasmon resonance (SPR) determination and photothermal detection. The optical sensing methods of *Salmonella* reported in recent years are shown in Table 2.

#### 4.1.1. Colorimetry

The most important feature of the colorimetric method is that the signal response can be observed by the naked eye. Meanwhile, the whole colorimetric experiment is simple, without the need for complicated instruments, so it is one of the ideal methods for a rapid in-field detection of *Salmonella* [56]. There are two main types of colorimetry: one is to produce a color change through the optical or chemical properties of the probe itself; the other is to obtain the color change of the chromogenic substrate through enzymatic or similar catalytic-like reactions. Here, classic studies were chosen to introduce these color-rendering methods.

**Table 2:** Optical sensors reported for *Salmonella* detection.

Detection Methods	Bioreceptor	Linear Range	Limit of Detection (LOD)	Detection Time	Real Sample Application	Reference
Colorimetry	Nucleic acid probe	100 to 10^9^ CFU/mL	16 CFU/mL	/	Milk	[57]
	Antibody	10^3^ to 10^8^ CFU/mL	10^3^ CFU/mL	14 min	Cabbage and drinking water	[58]
	Antibody	1.88 × 10^4^ to 1.88 × 10^7^ CFU/mL	1.88 × 10^4^ CFU/mL	/	Milk	[59]
	Antibody	0 to 10^8^ CFU/mL	500 CFU/mL	60 min	Milk	[60]
	Antibody	0 to 10^7^ CFU/mL	34 CFU/mL	/	Milk	[61]
	Antibody	100 to 10^4^ CFU/mL	100 CFU/mL	90 min	Milk	[62]
Fluorometry	Antibody	5 × 104 to 10^7^ CFU/mL	5 × 10^3^ CFU/mL	12 min	Broth	[9]
	Antibody	500 to 5 × 10^7^ CFU/mL	60 CFU/mL	60 min	Milk	[63]
	Antibody	40 to 4 × 10^6^ CFU/mL	40 CFU/mL	120 min	Chicken	[64]
	Aptamer	10 to 10^7^ CFU/mL	10 CFU/mL	/	Meat, milk and chicken	[65]
	Aptamer	50 to 10^6^ CFU/mL	35 CFU/mL	/	Chicken and shrimp	[66]
	Aptamer	12 to 5 × 10^5^ CFU/mL	11 CFU/mL	/	Milk	[67]
SERS	Aptamer	10 to 10^4^ CFU/mL	4 CFU/mL	/	Chicken and milk	[68]
	Nucleic acid probe	27 to 2.7 × 10^6^ CFU/mL	27 CFU/mL	30 min	Milk, chicken breast and beef	[69]
	Aptamer	100 to 10^7^ CFU/mL	50 CFU/mL	/	Milk	[70]
	Lectin	10 to 10^4^ CFU/mL	10 CFU/mL	/	/	[71]
	Aptamer	0 to 10^7^ CFU/mL	25 CFU/mL	/	Milk, orange juice, and tap water	[72]
SPR	Nucleic acid probe	0.01 to 100 ng/mL	10 pg/mL	60 min	/	[73]
	Antibody	/	7.4 × 10^3^ CFU/mL	80 min	Cucumber and hamburger	[74]
	Antibody	100 to 10^6^ CFU/mL	10^3^ CFU/mL	60 min	Powdered milk	[75]
Photothermal	Antibody	300 to 10^3^ CFU/mL	300 CFU/mL	90 min	/	[76]
	Antibody	100 to 10^7^ CFU/mL	100 CFU/mL	25 min	Milk and grape juice	[77]
	Antibody	10^4^ to 10^8^ CFU/mL	10^4^ CFU/mL	20 min	Milk and grape juice	[78]
	Antibody	10^3^ to 10^9^ CFU/mL	10^3^ CFU/mL	15 min	Milk and grape juice	[79]
	Antibody	5 to 5 × 10^3^ CFU/mL	70.7 CFU/mL	36 min	/	[80]

The commonly used colorimetric materials in non-enzymatic colorimetry are gold nanoparticles (Au NPs), which are excellent materials for identifying the presence of the target based on its wine red and obvious blue-purple color after aggregation. The antibodies and aptamers adsorbed on the surface of Au NPs prevent the surface charge of Au NPs from being destroyed by the salt solution, which slows down the solution color change caused by the aggregation of Au NPs [81]. Yi et al. developed an agglomerated Au NP colorimetry for *Salmonella* detection based on an aptamer. The aptamer was immobilized on chitosan and bonded to the surface of the Au NPs by electrostatic adsorption, and then the *Salmonella* combined with the aptamer and led to the loss of protection of aptamer for Au NPs. With the addition of the salt solution, the agglomeration of Au NPs would result in a color change, which can be recorded by an ultraviolet spectrophotometer for *Salmonella* detection. Moreover, the recoveries of *Salmonella* from spiked milk samples, between 92.4 to 97.2%, also confirmed the feasibility of this method in actual sample detection [57]. Among the paper-based colorimetric detection methods, the lateral flow test strip has always been the most popular detection platform. Based on the van der Waals forces of *Salmonella* on agglomerated Au NPs, Ren et al. realized the label-free sensitive determination of *Salmonella* by the naked eye through an immunochromatographic strip. The visual LOD of 10^3^ CFU/mL based on salt-induced aggregated Au NPs in the developed immunochromatographic strip was 100-fold lower than the method based on cationic AuNPs, and the test strip also exhibited an excellent recovery in cabbage and other food samples [58]. In addition to Au NPs, many kinds of nanomaterials with conspicuous colors make a great contribution to the colorimetric detection of *Salmonella* [59]

**Figure 2:**
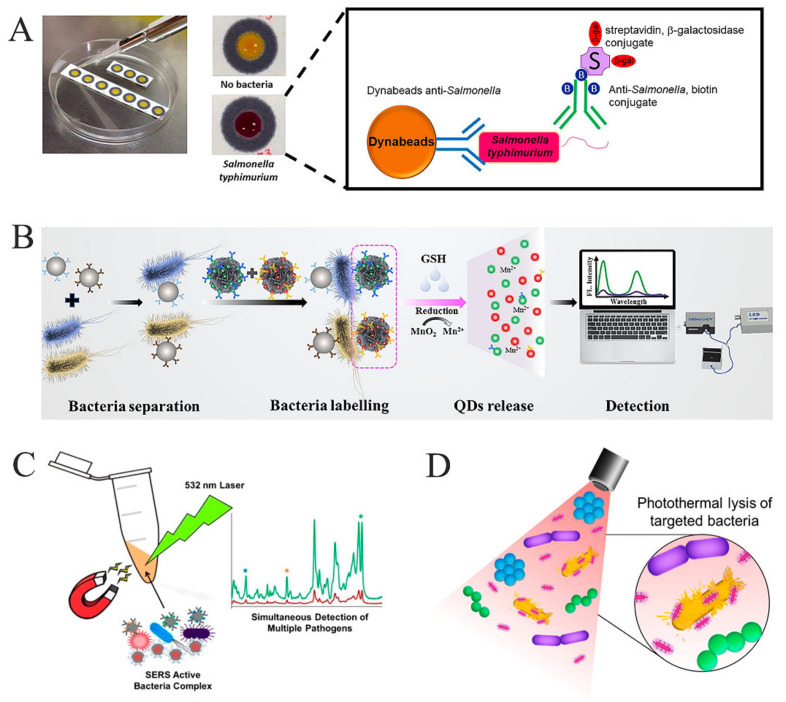
Optical sensing for *Salmonella* detection. (**A**) A paper-based colorimetric detection device [62]. (**B**) Fluorescence immunoassay for *Salmonella* by the loading and release of quantum dots [64]. (**C**) Label-based signal detection strategy based on the surface-enhanced Raman spectral scattering [71]. (**D**) A sensing strategy of targeting lytic *Salmonella* by the photothermal effect of Au NPs [80].

The other colorimetric method is an enzymatic or enzyme-like catalytic reaction based on ELISA, and the chromogenic substrate used is generally tetramethylbenzidine (TMB). The color of TMB from colorless to blue catalyzed by horseradish peroxidase (HRP) is a classical colorimetric method for *Salmonella* detection. Chen et al. enriched *Salmonella* by magnetic separation and then used Au NPs and HRP to catalyze TMB for signal enhancement, which was used for the specific determination of *Salmonella* in a food matrix [60]. Due to the low stability, low environmental tolerance and high cost of HRP, many mimic enzymes have been used in the colorimetric detection of *Salmonella* after Fe_3_O_4_ nanoparticles proved with peroxidase-like activity [82]. Cheng et al. synthesized a Fe-MOF nanozyme, which remained in the solution after magnetic separation was used to catalyze TMB for the *Salmonella* colorimetric analysis. The use of a nanometer enzyme caused the LOD of *Salmonella* to be as low as 34 CFU/mL, with a good storage stability and detection potential in milk samples [61]. Besides Fe-based nanozymes, graphene, molybdenum disulfide and other nanomaterials also play an important role in the study of the colorimetric detection of *Salmonella* by catalyzing TMB. In addition to TMB, there are a variety of chromogenic substrates for *Salmonella* detection. Srisa-Art et al. developed a paper-based colorimetric device combined with magnetic separation to visually detect *Salmonella* using chlorophenol red-β-d-galactopyranoside colorimetry based on β-galactosidase (Figure 2A). The white paper provides a good background for color generation, which obtained a LOD of 100 CFU/mL without any complex processing or enrichment of the samples, and has a good performance in whole milk [62]

With the development of detection technology and the more intelligent portable equipment, the colorimetric analysis method that judges the bacterial residue in foods according to the color change has become increasingly popular. The real-time monitoring of *Salmonella* is an important means to ensure the safety of consumers. The colorimetric method with a convenient measurement and simple operation is undoubtedly convenient for sampling inspections by non-professionals, which are of great value for food safety prevention and control in remote areas. However, the interference of the background of food samples in the colorimetric method is also a difficult problem that affects its accuracy, and hence a challenge that needs to be faced.

#### 4.1.2. Fluorescence Analysis

Fluorescence analysis is one of the optical sensing methods to quantify the concentration of the target according to the fluorescence intensity of the labeled materials, which can solve the color interference of a food matrix when using colorimetric methods. The generated fluorescence signal can be visually observed by a fluorescence observer or measured by a fluorescence spectrometer [83,84]. Fluorescence analysis has two main means of fluorescence generation; one mean is the direct measurement of the fluorescence intensity from fluorescent materials; the other mean is the indirect measurement of fluorescence quenching to reflect the amount of *Salmonella*.

The detection signal of sandwich fluorescence sensing is directly obtained from a fluorescent material such as quantum dots (QDs), which is the most common fluorescence detection method for *Salmonella* [85]. QDs with a high fluorescence yield and a long fluorescence lifetime have always been popular in fluorescence sensors. Hu et al. developed lateral flow immunoassay strips based on the silicon shell protected quantum dot nanospheres and realized the rapid detection of *Salmonella*. The whole detection process can be completed in 10 min, and obvious fluorescence signals can be observed with the naked eye, which provides a new opportunity for the prevention and control of *Salmonella* in foods [9]. Immunomagnetic beads are also commonly used for sample concentration to improve the detection sensitivity of fluorescence analysis. In the work of Yin et al., the immunomagnetic beads and QDs were combined to realize the low background and high sensitivity detection of *Salmonella* through the reverse assaying strategy, and the LOD could reach 60 CFU/mL, which led to a 50-fold improvement of the sensitivity compared with a conventional QD-based immunosensor. Moreover, the immunosensor can also achieve an accurate quantification of *Salmonella* in milk samples within 1 h [63]. Xue’s group proposed an innovative work: they loaded QDs with MnO_2_ nanoflowers and separated the part that bound to the bacteria by immunomagnetic beads. Then, the addition of glutathione reduced MnO_2_ to Mn^2+^, which caused the QDs to be released. The LOD of *Salmonella* with 40 CFU/mL was obtained by measuring the fluorescence intensity of the released QDs, and the method was successfully verified in chicken samples (Figure 2B) [64]. More and more fluorescent materials with excellent performance have been used in *Salmonella* detection through sandwich-format-based fluorescence analysis, such as upconversion nanoparticles, organic fluorophores, fluorescent microspheres and so on [86,87].

The fluorescence analysis methods designed based on fluorescence resonance energy transfer (FRET) are also popular [65]. Compared with sandwich fluorescence sensing, FRET can achieve a signal output without washing steps. “Turn-off” and “turn-on” are the two detection models of fluorescence signal in FRET. The “turn-off” model relies on the close proximity of fluorescent materials and the quencher in the process of target recognition, which leads to FRET and weakens the fluorescence signals, while the “turn-on” model realizes the signal measurement through the fluorescence recovery caused by the fluorescent materials being separated from the fluorescent acceptors after being combined with the target. Fluorescence observation with a dark background in FRET may be a more sensible choice due to its better signal-to-noise ratio, which may also be the reason for the higher number of studies on the “turn-on” model [88]. Duan et al. prepared a fluorescent acceptor by modifying the aptamers on the surface of QDs and blocking the fluorescence emission using carbon nanoparticles. In the presence of *Salmonella*, QDs separated from the carbon nanoparticles through the aptamer-bacteria recognition and re-emitted fluorescence. At this point, the fluorescence intensity of the solution reflected the amount of *Salmonella*, and the good practicability and stability of this method were also confirmed in shrimp and chicken samples [66]. Cheng at al. have synthesized a TM upconversion nanoparticle by doped Mn^2+^ into NaYF_4_: Yb and used the “turn-on” model, which constructs a fluorescence donor with the emission peak at 807 nm and, subsequently, a couple of aptamers. Then, the Au nanorods can lead to the fluorescence quenching of upconversion nanomaterials, while the presence of *Salmonella* prevented the energy transfer and the fluorescence recoveries. The application performance of the method has been verified in milk samples, and the LOD was as low as 11 CFU/mL under optimal conditions [67].

Fluorescence analysis can obviously solve the problems of color interference in the food matrix and has a lower detection limit, which is important for the detection of traces *Salmonella* compared with colorimetric detection. However, although the ubiquity of portable fluorescence detectors has accelerated the commercialization of fluorescence analysis, its inherent disadvantages are also obvious. The uncontrollable fluorescence bleaching caused by complex food substrates has always been a great problem in fluorescence analysis, and the application of new materials with better stability and optical properties is also a key measure to solve the pain point.

#### 4.1.3. Surface-Enhanced Raman Spectroscopy Detection

The Raman spectrum is a scattering spectrum which provides unique fingerprint information on different substances according to molecular vibration, and the Raman shift can describe the spectral information of different molecules [68]. SERS is an optical sensing technology that uses roughened metal surfaces or special nanomaterials to enhance Raman scattering and improve the detection sensitivity. Precious metals are the classic materials used for SERS, in which taking silver and gold as enhanced substrates is the most interesting option [69]. The earliest SERS for *Salmonella* detection can be traced back to 2003, with the unsatisfactory LOD of 10^6^ CFU/mL, which was limited by the detection equipment and single colloidal gold particles. However, the latest research progress has been beyond comparison [89]. Ma et al. designed a dimer probe that combined aptamer-modified Au NPs with silver nanoparticles. Then, *Salmonella* was enriched by magnetic beads and identified by the dimer probe to form a sandwich complex. The enhanced Raman scattering induced by gold and silver nanoparticles significantly reduced the LOD of *Salmonella* to 50 CFU/mL, and the method not only had a good selectivity for *Salmonella* but also confirmed its actual detection performance in milk samples [70]. Compounds with specific Raman shifts have also been used for *Salmonella* detection. Three kinds of pathogenic bacteria have been detected simultaneously by Kearns et al. using the SERS strategy based on magnetic separation. As shown in Figure 2C, magnetic materials coated with silver-binding lectin provided support for bacterial isolation and the enhancement of the Raman scattering, while a 7-dimethylamino-4-methylcoumarin-3-isothiocyanate binding antibody provided the specific signal production site of *Salmonella*. This method can not only realize the simultaneous determination of different pathogens in the same matrix, but also obtain a lower LOD, of 10 CFU *Salmonella* per mL [71].

Compared with the free precious metal substrate, the fixed precious metal surface also shows a promising Raman scattering enhancement effect to detect *Salmonella*. Using aptamer-modified silver nanorod arrays as substrates, Chen et al. successfully found that the Raman signals of *Salmonella* were different from the blank and control groups, which realized the signal amplification of *Salmonella*. SERS-active silver nanorod array substrates can amplify the specific Raman signals of *Salmonella* captured by the aptamer, and the existence of *Salmonella* can be verified according to noticeable spectral changes [90]. In addition, the vancomycin-coated silver nanoparticle array designed by Liu et al. also provides a reference for SERS analysis of *Salmonella* in foods [91]

SERS detection can obtain accurate *Salmonella* detection results with a lower amount of sample consumption, which has a good application prospect in the prevention and control of food safety. However, the sensitivity of the miniaturized Raman detector is not satisfactory, which is also a worrying aspect of SERS detection.

#### 4.1.4. Surface Plasma Resonance (SPR) Determination

The SPR sensor, a kind of optical sensor, can monitor multi-component interactions in real time and in situ through refractive index changes without labeling [92]. The basis of SPR is the sensing interface composed of a metal substrate. Similar to SERS, the materials dominate the detection performance of SPR, and the classic gold chips is also the preferred substrate for SPR [73]. Singh et al. presented an SPR nucleic acid sensor constructed by fixing ssDNA on the surface of the gold plate, which has carried out a sensitive, label-free and real-time monitoring of *Salmonella* conservative genes [93].

The label detection of *Salmonella* by SPR is also developing rapidly [94]. The SPR labeling detection designed by Vaisocherová-Lísalová et al. used Au NPs as the labeling material, and then the thickness of substrate materials was studied to evaluate the recognition ability of the sensor. The acceptable LOD of *Salmonella* at 7.4 × 10^3^ CFU/mL in cucumber was acquired by virtue of the superior surface resistance after Au NPs labeling. However, such sensitivity has great limitations in actual detections, which is not conducive to the rapid identification of low concentrations of *Salmonella* [74]. Enhancing the detection signal through an enzyme-catalyzed reaction in SPR has also been developed. Farka et al. used HRP-conjugated secondary antibodies as *Salmonella* markers to improve the detection sensitivity by catalyzing the conversion of 4-chloro-1-naphthol into insoluble precipitates. The sensitivity of this precipitation enhancement method was 40 times higher than that of the label-free approach. However, the LOD of 10^3^ CFU/mL in powdered milk is still lower than that of fluorescence and Raman detection [75].

The low sample consumption and real-time monitoring of SPR attracted investors in the commercialization process. Although SPR sensor miniaturization has been achieved, it still faces a low sensitivity. How to improve its stability in sample testing and the auxiliary of the precision instrument are the questions that researchers should think about.

#### 4.1.5. Photothermal Detection

Photothermal detection is an innovative and rapidly developing method for the detection of contaminants in recent years [95,96]. When some nanomaterials are irradiated by photon energy with different wavelengths, the vibration inside the atom converts light energy into heat energy, resulting in a photothermal effect, and this is the cornerstone of photothermal detection [97]. Common nanomaterials such as Au NPs, graphene oxide, black phosphorus, molybdenum disulfide and Prussian blue are used in photothermal detection frequently [98,99,100,101,102].

In general, photothermal detection shows the concentration of the targets through the photothermal temperature of the intercepted nanomaterials. The Au NPs with good photothermal properties are commonly used in immune analysis. The first study on improving the sensitivity of colloidal gold test strips by photothermal detection was carried out by Qin et al. [103]. This motivated the use of the photothermal properties of colloidal gold in many rapid detection methods. Especially in Zhang’s group, a variety of immunochromatographic test strips assays based on photothermal nanomaterials have been developed for *Salmonella* detection in recent years. They performed photothermal tests on commercial test strips based on Au NPs using a self-built portable sensor device to confirm the feasibility of improving the sensitivity of the test strip [104]. In order to achieve a more sensitive *Salmonella* detection, more and more nanomaterials with a higher photothermal conversion efficiency have been used in rapid detection methods to amplify the detection signal. The immunomagnetic beads which can enrich bacteria were firstly selected. Zhang et al. realized the capture, detection and killing of *Salmonella* using the immunomagnetic beads in 1.5 h, and a LOD as low as 300 CFU/mL was obtained. Then, they verified the detection performance of this method in drinking water, which provides a reference for the development of *Salmonella* detection methods in food [76]. Lu et al. not only used MoS_2_@Au nanomaterials with a higher photothermal conversion efficiency, but also oxidized TMB through its good catalytic performance. The nanomaterial and oxidized TMB were combined for photothermal detection, which made the LOD of *Salmonella* reach 100 CFU/mL. In addition, the application of this method has been validated in milk and grape juice samples, and the detection time of less than 25 min also shows its good prospect in the detection of *Salmonella* in foods [77]

Besides photothermal materials, the choice of temperature reading device is also related to the convenience and sensitivity of the methods. The thermal imager commonly used in the above methods is expensive, bulky and often used with a computer, which limits its popularity [105]. Therefore, many new works highlight the low cost and practicality of temperature reading devices. A card reader designed and manufactured by Zhang et al. has specially been used for the test strip to further increase its quantitative sensitivity, which is a strong boost to the standardization and commercialization of rapid photothermal detection [104]. Wang et al. focused on the detection of *Salmonella* in the changing environment using a resistance temperature sensor in a graphene oxide-based immunofiltration strip. The small volume of the sensor and the short detection time (within 20 min) contributed to the field prevention and control of *Salmonella* [78]. The following work innovatively integrated the temperature reading device and detection carrier, which greatly simplified the detection process. Du et al. immobilized the anti-*Salmonella* antibody on the mercury head of a glass thermometer, and the bacteria in the test solution was recognized by immune-graphene and then connected to the surface of the mercury head based on a sandwich immunoassay. The mercury head combined with immune-graphene was irradiated with laser light, and the temperature change can be shown directly on the thermometer. The 15-min detection time and the successful detection of *Salmonella* in milk and grape juice samples indicates the good application potential of this novel immune-thermometer. However, this novel method is also faced with the limitation of its low sensitivity (LOD of 10^3^ CFU/mL), which needs to be improved [79]. Instead of using laser irradiation to trigger the photothermal temperature, Guo et al. developed a new temperature generation strategy [106]. They designed nanomaterials with the activity of peroxidase to decompose hydrogen peroxide and produce oxygen, which forced the pre-placed calcium oxide to react with water and generate heat by increasing the pressure in the space. Then, the temperature change was measured by a temperature sensor carried on the mobile phone to judge the number of bacteria. This temperature generation strategy made the detection more automatic and reduced the manual operation steps. Although this work is not a temperature detection strategy triggered by a laser, this temperature detection method is coincident with photothermal detection.

Aside from the direct measurement of the photothermal temperature, the photothermal effect is also applied to *Salmonella* detection in other forms. Kim et al. used the photothermal temperature of gold nanorods to lytic bacteria directionally, and then measured the adenosine triphosphate bioluminescence in the lysis bacteria for a qualitative and a quantitative analysis of the pathogenic bacteria (Figure 2D). In this study, the photothermal effect was used for bacterial lysis, which greatly simplified the operation process and achieved a low LOD of 70.7 CFU/mL in 36 min. Moreover, the successful application of this method on plastic cutting boards also indicates its application potential in food sample detection [80]

With the development of photothermal detection, the more intuitive display of target concentration becomes more and more unique, which provides a new idea for the photothermal detection of *Salmonella*. Despite more than a decade of development, photothermal detection still has a long way to go before its formal commercialization, which is limited by its instability. The addition of more stable photothermal materials and the development of accurate detection instruments are crucial for its follow-up development.

### 4.2. Electrochemical Detection

Electrochemical sensing has always played an important role in bacterial detection due to its extremely low LOD and high application potential [107]. Electrochemical identification is mainly divided into voltammetry and impedimetry. Table 3 summarizes the electrochemical detection methods of *Salmonella*.

#### 4.2.1. Voltammetry

Voltammetry has always been a hot topic in electrochemical detection, and is still enduring after decades of development [119,120]. Voltammetry generally monitors current changes by applying different voltages to reflect the analyte concentration. There are three main types of voltammetry sensors for the monitoring of *Salmonella* in foods: cyclic voltammetry, differential pulse voltammetry and square-wave anodic stripping voltammetry. Silva et al. used ethylenediaminetetraacetic acid disodium salt as the reducing agent to form Au NPs on a polyvinyl chloride (PVC) membrane to construct a polymer electrode. The design of the polymer membrane significantly amplified the electrochemical signals and confirmed the *Salmonella* residues through the potential shift caused by the recognition of antigens and antibodies. What is impressive is that the polymeric ion selective electrodes can achieve an astounding LOD of 6 *Salmonella* cells within 1 h and were validated in apple juice samples [108].

The use of metal electrodes and enhancing electrical signals by depositing nanomaterials is also popular. Singh et al. deposited the composite of graphene oxide and multiwalled carbon nanotubes (GO-cMWCNTs) on an indium tin oxide electrode and then sealed the aperture with polydimethylsiloxane. The GO-cMWCNTs composited on the electrode provided sufficient antibody loading sites and a high electron transfer rate to the electrode, which made the recognition performance of *Salmonella* reach 0.376 CFU/mL. Such a low LOD for this method can detect *Salmonella* contamination earlier and respond effectively [109]. Nguyet et al. synthesized core-shell cerium oxide nanorods for electrode modification and covalently attached ss-DNA to the surface of the microelectrodes. The nucleic acid probe biosensor has obtained a LOD of 0.084 nM due to the deposition of the core-shell nanorods [121].

While a label-free electrochemical detection shows great allure, voltammetric sensors labeled with enzymes or nanomaterials are also personally involved in improving sensitivity [122,123]. The Au NPs deposited on the screen-printed electrode were used for enhancing the electrochemical signal and connecting antibodies to capture *Salmonella* in Fei et al.’s work. Then, HRP-linked secondary antibodies were added to form a sandwich structure, and the signal of newly added thionine oxidized by HRP was measured by cyclic voltammetry. The number of bacteria, in the range of 10^4^ to 10^6^ CFU/mL, showed a good linear relationship with the intensity of the electrical signals, and the LOD was 3.0 × 10^3^ CFU/mL [110]. In addition, Freitas and Viswanathan et al. also detected *Salmonella* by labeling cadmium sulfide and copper sulfide on the surface of *Salmonella*, respectively. With the enhancement of the electrical signals by the two compounds and the use of square-wave anodic stripping voltammetry, they obtained low detection limits of 13 cells/mL and 400 cells/mL, respectively, and the suitability of the methods in the determination of real samples has been tested in milk samples [111,112].

Voltammetry, one of the pioneers of electrochemical detection, has experienced many vicissitudes in life, but still faced threats such as the stability of electrodes and labeling materials. The electrical activity of the detection electrode and the stability of the label material always restrict the further upgrading of voltammetry. Nowadays, the rapid development of biometric technologies and advanced nanomaterials promotes the progress of voltammetry, which is expected to become the normative standard for high sensitivity and specificity detection of *Salmonella* in foods in the future.

#### 4.2.2. Impedimetry

**Figure 3:**
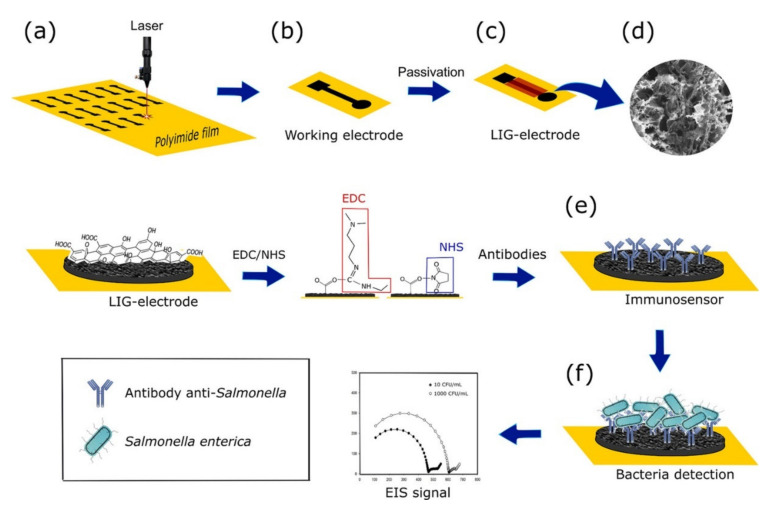
Schematic diagram of a label-free electrochemical immunosensor for *Salmonella* detection. (**a**) label-free laser-induced graphene processing, (**b**) working electrode, (**c**) passivation of the working electrode, (**d**) SEM image showing, (**e**) antibodies immobilization, (**f**) *Salmonella* binding to the electrode [115].

Impedance, expressed in terms of current-voltage ratio, reflects the resistance of the circuit under the change of electric field, and is generally measured by electrochemical impedance spectroscopy (EIS) [124]. The ultra-low LOD provided by impedance biosensors is outstanding, and one of the main reasons for its rapid development. Impedance biosensors generally consist of a three-electrode system (working electrode, auxiliary electrode and reference electrode), which can realize label-free detection, although label detection is also very important for improving sensitivity [119]. For the sake of improving the sensitivity, Bagheryan et al. fixed the diazonium-supporting layer to the electrode and connected the amino-modified aptamer to the surface of the electrode for capturing *Salmonella*. The LOD of the impedance biosensor can reach an amazing 6 CFU/mL by a denser aptamer layer formed on the electrode after the immobilization of the diazonium-grafting layer. Moreover, the good recovery of the sensor in an apple juice sample also demonstrated its applicability for real sample detection [113]. The work of Ranjbar et al. has lowered the LOD of *Salmonella* to the extreme. By electrochemical synthesis, they synthesized porous gold on the surface of a gold electrode and immobilized the aptamer to capture *Salmonella*. The nano-porous gold synthesized by an Au-Cu alloy greatly improved the detection sensitivity of the method, which can lower the LOD of *Salmonella* to 1 CFU/mL. Furthermore, the designed aptasensor can be successfully applied to the detection of egg samples and be capable of distinguishing living and dead bacteria [114]. In addition, the modification of electrodes by other nanomaterials with excellent electrochemical properties has also greatly promoted the improvement of the detection sensitivity (Figure 3) [115,116].

Label detection is also popular in the construction of impedance sensors. In the work of Xue et al., a specific ring magnetic bead network was formed using the immunomagnetic beads under the action of a high gradient magnetic field, and the *Salmonella* bacteria were captured in the specific location of the network. Then, the antibody modified MnO_2_ nanoflowers were added. After the formation of the three-layer sandwich structure, the resistance was increased by the injection of hydrogen peroxide and then reduced by the production of Mn^2+^ decomposed from MnO_2_. The addition of a microelectrode made the impedance measurement more convenient, and the LOD of 19 CFU/mL was obtained. The *Salmonella* capture efficiency of this method was more than 60%, which also provided a feasible scheme for direct bacteria separation from large-volume food samples [117]. Wang et al. also used hydrogen peroxide to reduce MnO_2_ to realize the impedance change of the solution to detect *Salmonella*, which abandoned the operation of modifying bioreceptors on the electrode. Magnetic separation was mediated by immunomagnetic beads and formed the complex with *Salmonella* and SiO_2_@MnO_2_ nanocomposites, making it fully applicable in milk samples. The chip designed in a lab also laid the foundation for the commercialization of impedance sensors [118].

The great success of impedimetry in the detection field is inseparable from its short time consumption, high sensitivity and miniaturized device. However, the differences in detection signals caused by different batches of electrodes and the uncertain status of the probes need to be improved. The development of new electrodes, the preparation of special materials and the new screen-printed means are undoubtedly huge pushes for the popularization of impedance sensors. In particular, the standardization and unification of impedance signals may be a fundamental turning point for improving the reliability of detection results.

### 4.3. Other Signal Transduction Methods

Other rapid *Salmonella* detection methods are also driving advances in *Salmonella* control in food, such as mass-based sensors and magnetic biosensors [125,126]. The piezoelectric biosensors and the magnetic relaxation switching will now be introduced.

#### 4.3.1. Piezoelectric Biosensors

The piezoelectric sensor depends on the piezoelectric effect generated by the external force on the dielectric of the sensor surface, and the detection signal is measured by the quartz crystal microbalance (QCM) [127]. For example, Fulgione et al. used QCM to obtain a LOD of *Salmonella* in chicken of less than 1 CFU/mL with a pre-enrichment step for 2 h, and the LOD in chicken samples was also less than 100 CFU/mL [128]. In recent years, there are relatively few studies on QCM for *Salmonella* detection, but its portability and real-time signal monitoring still require further attention.

#### 4.3.2. Magnetic Relaxation Biosensors

A magnetic relaxation biosensor is a kind of magnetic sensor, which is a method to detect the concentration of targets by the changes of the relaxation time of magnetic materials in nuclear magnetic resonance (NMR) [129]. Magnetic relaxation sensing generally uses transverse relaxation time (T_2_) to judge the amount of *Salmonella* [130]. Wang et al. designed a paramagnetic ion-mediated magnetic relaxation sensor based on Mn(VII)/Mn(II) interconversion. Alkaline phosphatase combined with immune-magnetic spheres was used for *Salmonella* separation, and then nonreducing ascorbic acid phosphate was catalyzed to ascorbic acid, which led to the reduction of Mn(VII). The change of T_2_ induced by the Mn(VII) reduction predicted the presence of *Salmonella* and could be numerically calculated, and the LOD was as low as 20 CFU/mL, with a good recovery of milk samples [131]. Apart from metal ions, hydrogels are also used for magnetic relaxation switching. Chen’s group evaluated *Salmonella* residues through the change of the T_2_ signal generated by the sol-gel transformation of mediated sodium alginate. This innovative study also rids the magnetic relaxation biosensor of the limitation of metal ions, and this method’s LOD of 50 CFU/mL and good applicability in milk samples also offers a brand-new platform for *Salmonella* detection in foods [132]. The high sensitivity and low operational difficulty of magnetic relaxation detection have opened a new door for *Salmonella* detection. However, the miniaturization and popularization of detection instruments are important ways of improving the recognition of this method.

## 5. Challenges and Trends of Rapid Detection Methods

The popular rapid detection methods for *Salmonella* detection in recent years and their shortcomings have been summarized and analyzed. Here, the reasons why many current rapid detection methods are difficult to use in *Salmonella* prevention and control were summarized, along with realistic problems that need to be solved. At present, the application obstacles of various rapid detection methods come mainly from the following aspects: complex sample pretreatment, low stability and difficulties of non-destructive testing and in-field application.

### 5.1. Sample Pretreatment

**Figure 4:**
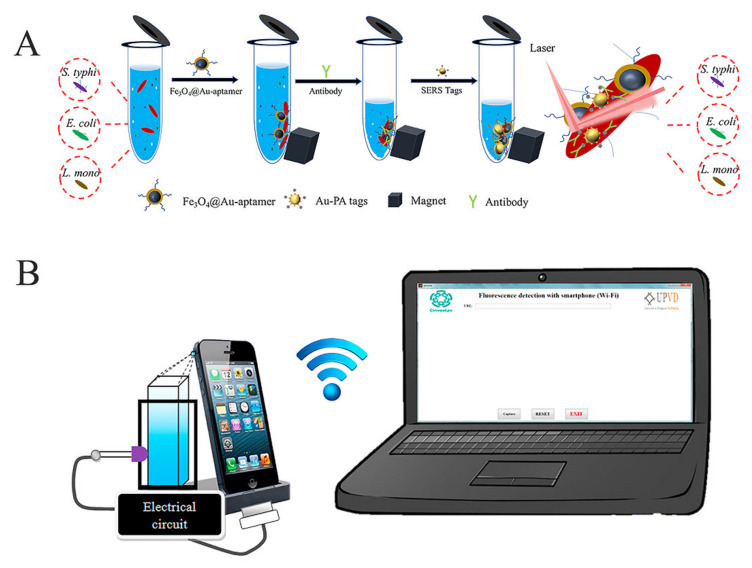
(**A**) Enhancement of specific and sensitive sensing of *Salmonella* by the pretreatment of magnetic separation and enrichment [72]. (**B**) Fluorescence detection strategy based on a smartphone [139].

The complexity of food matrixes is unmatched by other actual samples (such as waste water and soil samples), and the reliable pretreatment schemes with less loss of targets are even more critical than the excellent detection methods. A qualified sample pretreatment can remove the complex components in food matrixes and enrich *Salmonella*, which is very important for the successful implementation of the subsequent detection process. Filtration and centrifugation are the earliest methods of *Salmonella* enrichment, which are simple and in-expensive [133]. But the non-specific enrichment easily misses the target bacteria and reduces the accuracy of *Salmonella* detection. Nowadays, many kinds of *Salmonella* sorting technologies have been developed, such as magnetic separation, electrophoresis, microfluidic screening and so on (Figure 4A) [72,134,135,136]. Immunomagnetic separation is the widely recognized method for the isolation and enrichment of *Salmonella*. However, in magnetic separation the aggregation and sedimentation caused by special salt ions in a food matrix need to be overcome. Therefore, the development of screening and identification methods integrating pretreatment and detection, such as microfluidic technology integrating target capture and signal transduction, is one of the ways to prevent and control *Salmonella* efficiently. Therefore, the developed methods with resistance to matrix interference can solve the above problems perfectly [137,138].

### 5.2. Non-Destructive Testing

The detection method that needs sample pretreatment indicates a destructive detection, which will undoubtedly damage foods and miss the targets. Especially for trace target detection, the rapidity and non-actual matrix dependence of non-destructive testing will reduce the difficulty of the testing operation, which can help manufacturers to control the cost and supply the products as soon as possible, also avoiding post-market recalls [140]. At present, non-destructive testing in foods mainly depends on spectral scanning, which is often used for the determination of food components and is difficult for pathogenic bacteria detection [141,142]. Moreover, it is hard to find the intrinsic harmful factors merely by detecting the shallow layer of food surface, which is also a great hidden danger. It is undoubtedly a subversive progress to develop automatic, intelligent and efficient non-destructive testing technology for pathogenic bacteria in foods [143,144].

### 5.3. In-Field Application and Stability Problems

The realization of real-time and in-field detection is the most significant application of the developed rapid detection methods. The most common limitation of advanced detection methods with ultra-high sensitivity are complicated and precise instruments, which are bulky and non-portable. Although simple and intuitive methods such as colorimetry are more suitable for in-field rapid screening, the low sensitivity is a major limitation. However, the development of portable instruments makes optical and electrochemical sensors more convenient to use. Furthermore, the stability of the biosensors may be more important for in-field detection, except for the dependence on instruments. The changing environmental temperature and complex food matrix seriously affect the performance of the sensors by impairing the function of bioreceptors (such as antibodies and other recognition proteins) [145]. To improve the detection stability, recognition elements with high anti-interference ability, such as aptamers, are widely used in the establishment of detection methods, which is also one of the mainstream research directions at present [146,147]. The broad way to accelerate the industrialization of rapid detection products is screening aptamers with high affinity and specificity. Meanwhile, the study of the recognition mechanism of aptamers is also of great significance.

In addressing practicality and stability, user-friendly *Salmonella* detection methods supported by functional nanomaterials, portable sensing platforms and advanced technologies also guide the future development. The development of new materials and nanotechnology is the key to signal generation and to promote the progress of the detection methods. Especially, materials (precious metal-based materials, carbon-based materials, metal organic frame materials, QDs, upconversion materials, etc.) with good optical, electrical and biological compatibility and enzyme activity simulation can greatly improve the sensitivity and stability of the sensor [148,149]. Even though the new materials have been applied in *Salmonella* detection explosively, improving the properties of materials is still the most relevant topic.

Nanomaterials are the source of signal transduction, but signal reception and processing could not be separated from various instruments. The volume and energy consumption of instruments are the main influencing factors of in-filed detection, so more portable and intelligent instruments are being commercialized. With the development of optical and electrochemical sensing technology, the instruments used in rapid detection are being miniaturized and rendered more accurate and portable to be used specifically in colorimetric, fluorescent, photothermal and electrochemical signal reading instruments [150,151]. A typical popular detection tool is a smartphone with an intelligent operating system, which has been used as an advanced sensing platform assembling optical and electrical sensing devices. The most intensively studied sensing strategies of smartphones are fluorescence detection and temperature measurement, which have achieved fruitful results in rapid detection (Figure 4B) [139,152].

In addition to signal collection, the choice of detection platform is also an important issue. ELISA and lateral flow test strips have been commercialized on a large scale and are the most recognized detection platforms [153,154]. New detection platforms such as microfluidic devices have also been actively developed. They amount to miniature laboratories that can integrate sample pretreatment, target separation and detection, which has the advantages of miniaturization, standardization and automation. Their modular detection steps and high-throughput screening properties are ideal choices for the in-field prevention and control of *Salmonella* [155,156,157].

## 6. Conclusions

The high incidence of *Salmonella* contamination in foods and its serious threat to human health have led to an explosive development of *Salmonella* rapid detection methods in a decade. The advantages and disadvantages of different bioreceptors for *Salmonella* recognition in rapid detection were discussed. With the development and utilization of various recognition receptors, the accuracy of rapid *Salmonella* detection methods has grown gradually closer to that of the traditional methods of culture and gene sequencing. Moreover, the diversity of recognition receptors have also broadened the design ideas of detection methods and accelerated the popularization of different sensing strategies. Then, the most popular methods for the rapid detection of *Salmonella* were summarized and classified according to different signal transduction mechanisms. The classical optical and electrochemical biosensors for *Salmonella* detection based on bioreceptors were reviewed in detail. Although colorimetry is easily conditioned by the color of the samples, it is still the simplest and most effective method for the preliminary identification of *Salmonella*. The high sensitivity of the fluorescence method depends on the fluorescence spectrophotometer under a high background noise from the matrix. The strong anti-matrix interference ability of SERS will evolve further after the development of more accurate and smaller instruments. However, the sensitivity of SPR needs to be improved, although it can achieve micro-detection and real-time monitoring. Due to the development of small-scale detection equipment and instruments, photothermal detection has recently created a new vision for *Salmonella* prevention and control. However, the unstable and insensitive detection results of photothermal detection are still the dominant limitations. The electrochemical biosensor with the ultra-high sensitivity is a practical detection method with a great potential. However, the complex food matrix is very troublesome for electrochemical biosensors, and the sample pretreatment must be sufficient.

The ideal rapid detection methods for *Salmonella* should have great resistance to the interference of the food matrix and a complex external environment for reducing the manual operation steps as much as possible. Sample pretreatment mainly includes target enrichment and separation, such as immunomagnetic separation, to guarantee the performance of the subsequent sensors. As a smartphone was successfully applied in the rapid detection, the miniaturization and automation of the detection equipment is also a major trend, which may be the most competitive detection strategy in the future. Restricted by the current detection technology, the combination of multiple sensing methods may be a more effective prevention and control strategy. In particular, a more attention-focused microfluidic technology can integrate sample processing, advanced nanomaterials and sensing devices to realize the innovative promotion of detection methods. The integration of new detection technologies for *Salmonella*, especially in the combination with information technology and big data analysis, needs to be further adjusted and improved to monitor *Salmonella* in real time in the whole food industry chain and provide an effective early warning for the relevant departments.

## Figures and Tables

**Table 1:** Summary of bioreceptors for *Salmonella* recognition.

Bioreceptors	Description	Advantages	Limitations
Antibody	Specific recognition proteins produced by immune cells stimulated by antigens	High affinity and specificity	Time-consuming and low output; poor resistant to high temperature and acid and alkali
Aptamer	A single stranded nucleic acid	Simple synthesis, strong anti-interference and high affinity	Special 3-dimensional structure is required to identify the target
Nucleic acid probe	Nucleotide sequences complementary to bacterial genes	Simple synthesis and high affinity	Special immobilization
Bacteriophage	A virus that can infect and replicate in bacteria	Identification of living and dead bacteria	Lysis of bacteria
Lectin	A class of non-enzyme and non-antibody proteins that can recognize carbohydrate chemicals	High stability and low cost	Limited selectivity, less practical types

**Table 3:** Electrochemical sensors reported for *Salmonella* detection.

Detection Methods	Bioreceptor	Linear Range	Limit of Detection (LOD)	Detection Time	Real Sample Application	Reference
Voltammetry	Antibody	13 to1.3 × 10^6^ cells/mL	6 cells/mL	60 min	Apple juice	[108]
	Antibody	10 to 10^7^ CFU/mL	0.37 CFU/mL	/	/	[109]
	Antibody	10^4^ to 10^9^ CFU/mL	3.0 × 10^3^ CFU/mL	/	Egg, chicken and meat	[110]
	Antibody	10^3^ to 5 × 10^5^ cells/mL	400 cells/mL	/	Milk	[111]
	Antibody	10 to 10^6^ cells/mL	13 cells/mL	60 min	Milk	[112]
Impedimetry	Aptamer	10 to 10^8^ CFU/mL	6 CFU/mL	/	Apple juice	[113]
	Aptamer	650 to 6.5 × 10^8^ CFU/mL	65 CFU/mL	/	Egg	[114]
	Antibody	10 to 10^5^ CFU/mL	13 CFU/mL	22 min	Chicken broth	[115]
	Antibody	10 to 10^7^ CFU/mL	10 CFU/mL	/	/	[116]
	Antibody	30 to 3.0 × 10^6^ CFU/mL	19 CFU/mL	90 min	Chicken	[117]
	Antibody	20 to 2.0 × 10^5^ CFU/mL	21 CFU/mL	/	Milk	[118]

## Data Availability

Not applicable.
